# Investigating the causal relationships between excess adiposity and cardiometabolic health in men and women

**DOI:** 10.1007/s00125-022-05811-5

**Published:** 2022-10-12

**Authors:** Pascal M. Mutie, Hugo Pomares-Millan, Naeimeh Atabaki-Pasdar, Daniel Coral, Hugo Fitipaldi, Neli Tsereteli, Juan Fernandez Tajes, Paul W. Franks, Giuseppe N. Giordano

**Affiliations:** 1 Genetic and Molecular Epidemiology Unit, Lund University Diabetes Centre, Department of Clinical Sciences, Clinical Research Centre, Lund University, Lund, Sweden; 2 Harvard T.H. Chan School of Public Health, Boston, MA USA

**Keywords:** Cardiometabolic, Causal, Mendelian randomisation, Obesity

## Abstract

**Aims/hypothesis:**

Excess adiposity is differentially associated with increased risk of cardiometabolic disease in men and women, according to observational studies. Causal inference studies largely assume a linear relationship between BMI and cardiometabolic outcomes, which may not be the case. In this study, we investigated the shapes of the causal relationships between BMI and cardiometabolic diseases and risk factors. We further investigated sex differences within the causal framework.

**Methods:**

To assess causal relationships between BMI and the outcomes, we used two-stage least-squares Mendelian randomisation (MR), with a polygenic risk score for BMI as the instrumental variable. To elucidate the shapes of the causal relationships, we used a non-linear MR fractional polynomial method, and used piecewise MR to investigate threshold relationships and confirm the shapes.

**Results:**

BMI was associated with type 2 diabetes (OR 3.10; 95% CI 2.73, 3.53), hypertension (OR 1.53; 95% CI 1.44, 1.62) and coronary artery disease (OR 1.20; 95% CI 1.08, 1.33), but not chronic kidney disease (OR 1.08; 95% CI 0.67, 1.72) or stroke (OR 1.08; 95% CI 0.92, 1.28). For cardiometabolic risk factors, BMI was positively associated with glucose, HbA_1c_, triacylglycerol levels and both systolic and diastolic BP. BMI had an inverse causal relationship with total cholesterol, LDL-cholesterol and HDL-cholesterol. The data suggest a non-linear causal relationship between BMI and blood glucose levels, HbA_1c_ and lipid fractions (*p*<0.001), more strongly in men than women. The piecewise MR results were consistent with the fractional polynomial results. The causal effect of BMI on coronary artery disease, total cholesterol and LDL-cholesterol was different in men and women, but this sex difference was only significant for LDL-cholesterol after controlling for multiple testing (*p*<0.001). Further, the causal effect of BMI on coronary artery disease varied by menopause status in women.

**Conclusions/interpretation:**

We describe the shapes of causal effects of BMI on cardiometabolic diseases and risk factors, and report sex differences in the causal effects of BMI on LDL-cholesterol. We found evidence of non-linearity in the causal effect of BMI on diseases and risk factor biomarkers. Reducing excess adiposity is highly beneficial for health, but there is greater need to consider biological sex in the management of adiposity.

**Graphical abstract:**

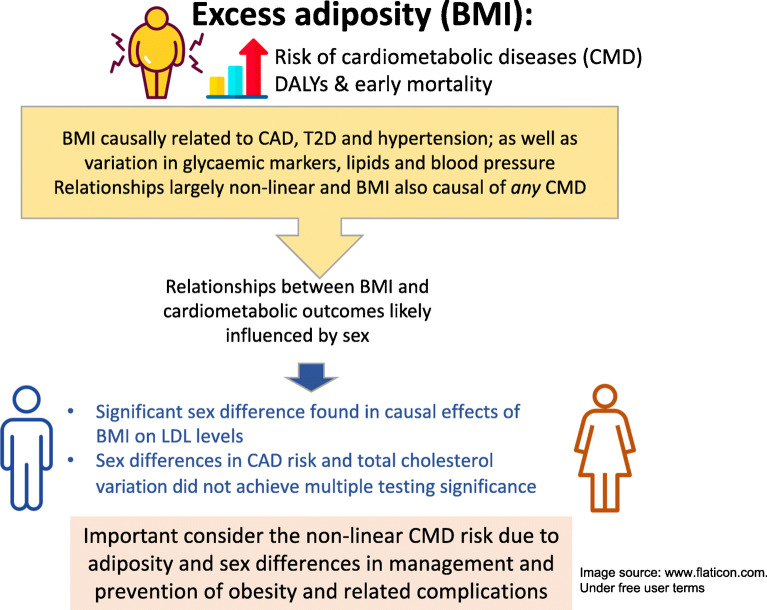

**Supplementary Information:**

The online version contains peer-reviewed but unedited supplementary material available at 10.1007/s00125-022-05811-5.

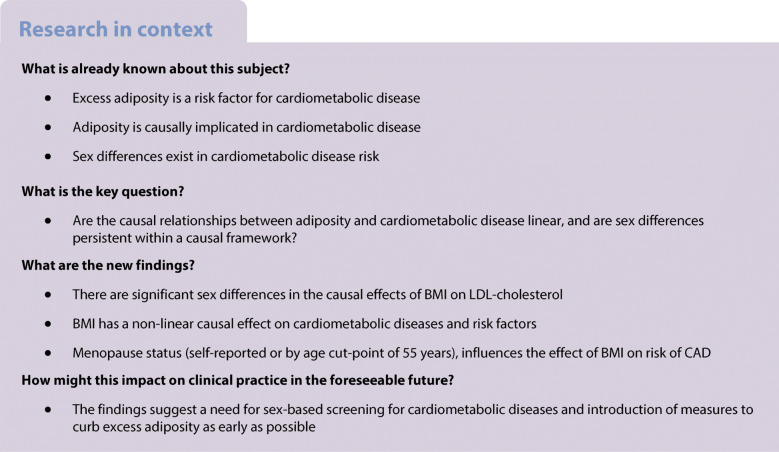



## Introduction

Cardiometabolic diseases (CMDs) are among the top ten causes of death and are associated with increased healthcare costs globally, making their relationship with adiposity a major public health concern [1–4]. Excess adiposity is associated with increased risk of CMDs, as well as increased risk of all-cause mortality [5–8]. The BMI category at lowest risk of early death is 20–25 kg/m^2^ in populations of European ancestry, with average health worsening significantly within the ‘overweight’ category, and deteriorating further as BMI increases [9]. Both observational studies and some causal inference studies suggest that BMI has a J-shaped relationship with all-cause and cardiovascular mortality [5, 10].

Observational studies often suffer from residual confounding and reverse causality, as do the relational shapes they describe. For example, the high mortality rate observed in some people with lower BMI (J-shaped relationship) is probably caused by the chronic disease cachexia [11]. While causal relationships between adiposity and CMDs have been determined previously, most studies assume these relationships are linear [12–15]. In addition, observational studies have shown that sex confers differential CMD risk profiles in men and women, but extensive investigation of such differences within a causal framework is lacking [16–19]. Therefore, understanding the nature of causal relationships between excess adiposity, CMDs and any sex differences therein may help to refine public health interventions [20].

Patterns of causal associations between excess adiposity and cardiometabolic outcomes remain understudied; given the shapes reported in observational studies, we hypothesised that adiposity has non-linear causal effects on cardiometabolic outcomes, with sex differences within this causal framework. The purpose of this study was to elucidate the nature of causal effects and explore the sex differences in the effects of BMI on CMDs (coronary artery disease [CAD], type 2 diabetes, chronic kidney disease [CKD], stroke and hypertension). We further extended these investigations to risk factor biomarkers: glycaemic markers (glucose, HbA_1c_), lipids (triacylglycerols, total cholesterol, LDL-cholesterol and HDL-cholesterol), lipoprotein A (LPA), urea and BP.

## Methods

### Population

We used individual-level data from the UK Biobank, a cohort of approximately 500,000 participants of mixed ancestries assessed across 22 centres in the UK. For this study, we selected individuals of white European descent only (*n*=409,584). In summary, participants aged 40–69 years were enrolled between 2006 and 2010, and standard anthropometric measurements were taken, in addition to biological samples (urine, blood and saliva); socio-demographic, lifestyle and other health determining factors were recorded. The UK Biobank study received approval from the Multi-centre Research Ethics Committee (reference 16/NW/0274), and all participants gave informed consent [21]. Information about recruitment and data collection has been provided elsewhere [22]. The current analysis is based on application number 57232 to the UK Biobank resource. Use of UK Biobank data for the analysis described here was approved by the Swedish Ethics Approval Authority (application number 2021-03174).

### Outcome variables

#### Disease outcomes

For each of the disease outcomes (type 2 diabetes, hypertension, stroke, CAD and CKD), information was obtained from ICD-9 (http://www.icd9data.com/2007/Volume1/default.htm) and ICD-10 (http://apps.who.int/classifications/icd10/browse/2016/en) diagnosis codes for both prevalent and incident disease, self-reported diagnoses, enrolment interview reports and self-reported medication data. We excluded participants whose reported age of type 2 diabetes diagnosis was 20 years old or less, as it was deemed to be probable type 1 diabetes. For cardiovascular disease (CAD, stroke and hypertension) and CKD, we additionally used information regarding surgical operations, plus interventional procedures related to each disease from OPCS4 codes (https://classbrowser.nhs.uk/#/), self-reported surgical procedures, and details of vascular diseases diagnosed by a doctor, which contained specific coding for each disease outcome. Additional information was obtained from medication data for both men and women, based on self-reported data collected at enrolment. Details of codes and data fields used for each disease are provided in electronic supplementary material (ESM) Table 1.

#### Disease-risk biomarkers

The disease-risk biomarkers that we included were glucose, HbA_1c_, triacylglycerols, cholesterol (total, HDL and LDL), urea and LPA, plus systolic BP (SBP) and diastolic BP (DBP). This information was obtained from the blood biochemistry categories of the UK Biobank, details of which are provided in ESM Table 2. For BP, we added 15 and 10 mmHg, respectively, to the values for SBP and DBP in participants taking BP medication [23].

### Genetic data

Details of enrolment and genetic data handling have been extensively explained by Bycroft et al [22]. For this project, we used version 3 of the imputed genotypes data from the UK Biobank. We excluded SNPs and individuals with a genotype call rate <99%, SNPs with a Hardy–Weinberg equilibrium *p* value <1×10^−10^, those with an imputation score <80%, any duplicated SNPs, and SNPs with a minor allele frequency <0.01. Using quality control results provided by UK Biobank, we further excluded individuals deemed outliers for heterozygosity (indicating poor sample quality or contamination), those with sex ambiguity and aneuploidy, and one of any pair of related individuals (up to third-degree relatedness, kinship coefficient 0.0442–0.0882). After further exclusion of participants with missing anthropometric measurements or HbA_1c_ beyond detectable ranges (>184 mmol/mol or 19%), our final sample comprised 333,582 individuals (ESM Fig. 1).

### Computing the BMI polygenic risk score

We used genome-wide association study (GWAS) summary statistics from the latest GIANT meta-analysis of BMI GWASs (excluding participants from the UK Biobank), and selected only genetic variants that were associated with BMI at a genome-wide significance level (*p*5×10^−8^): *n*=1560 SNPs [24]. Individual genetic data were obtained from the UK Biobank. A BMI polygenic risk score (PRS; PRS_BMI_) was calculated by weighting each SNP by its effect size from GWAS summary data and then summing these values for all SNPs for each individual in our sample. Prior to PRS_BMI_ calculation, clumping restricted to *r* ^2^=0.2 and a 250 kb window was performed to ensure that only SNPs that are not in linkage disequilibrium were used. After this quality control step, there were 89 uncorrelated BMI SNPs available for use in generating the PRS_BMI_. All PRS_BMI_ calculations were performed using PRSice-2 software [25]. To reduce the chances of horizontal pleiotropy between PRS_BMI_ and the various diseases and risk factors, we selected BMI SNPs specific to each trait. This was done by excluding any SNPs that were associated with the respective trait at genome-wide significance from the BMI SNPs by comparing with GWAS summary data for the trait. We then computed a trait-specific PRS_BMI_ (for instance, a PRS_BMI_ for CAD analysis that used BMI SNPs that were not associated with CAD) for use in downstream analyses involving that specific trait.

### Statistical analysis

#### Causal effect assessment

We used two-stage least-squares (2SLS) Mendelian randomisation (MR), with PRS_BMI_ as the genetic instrumental variable, to estimate causal effects of BMI on cardiometabolic traits. Prior to analysis, BMI was transformed in the same way as in the discovery GWAS by Locke et al [24]. Specifically, the effects of age, age squared, smoking status, alcohol consumption, UK Biobank assessment centre and the Townsend Deprivation Index were regressed out separately for men and women. Residuals from each of the models, men and women, were then inverse normal-transformed to create a main exposure variable representing BMI.

In the first stage, the exposure was regressed on the PRS_BMI_ in a linear model, adjusting for genotyping array and the first ten genetic principal components characterising the population substructure. Thereafter, fitted values were generated and used in the second stage of 2SLS, where logistic and linear regression models were used for binary and continuous traits respectively, with the fitted values as the exposure, adjusting for the same covariates as in the first stage. The regression coefficients of these fitted values in the second stage represent an estimate of the causal effect of BMI on the outcome [26]. We ran 2SLS models for each disease outcome and each biomarker, and also performed sex-stratified analyses.

Continuous outcomes were scaled so that the results represent a change in SD units of outcome per unit change in BMI. Cochran’s *Q* test was used to assess sex differences in the sex-stratified analysis. To estimate the causal effect of BMI on any CMD, we used both fixed and random effects meta-analysis, and considered the combined outcome as the likelihood of any CMD. We performed 15 main hypothesis tests (for the five disease outcomes and ten disease-risk biomarkers) and 30 sex-stratified tests; therefore, the Bonferroni-corrected significance level was set at *p*=0.001 (0.05/45).

#### Determining the shape of the causal relationships

To describe the shape of the causal relationships between BMI and each of the traits, we used a non-linear MR fractional polynomials method [27]. This involves calculating the local average causal effect (LACE) in quantiles of the exposure. These LACE estimates are then meta-regressed against the means of the exposure in each quantile, and tests of non-linearity are applied to test the null hypothesis that the resultant non-linear model is no different from a linear model. Given that stratifying directly on the exposure can lead to collider bias [27], we used two methods to construct these quantiles: the residual [27] and the doubly ranked [28] methods. In the residual method, the exposure is regressed on the genetic instrument, and the quantiles are derived from the residuals of this regression. While this ensures that the strata are independent from the genetic instrument, it has the caveat that it assumes homogeneity in the relationship between the genetic instrument and the exposure [28]. In the doubly ranked method this issue is addressed through a two-step process. First, individuals are categorised into pre-strata according to their level of the genetic instrument. Subsequently, within each of these pre-strata, individuals are ranked based on the level of the exposure. The final quantiles are then constructed by selecting individuals with equal ranks in the pre-strata, thus making the distribution of the genetic instrument similar across the final quantiles, while ensuring that the average level of the exposure is increasing across these final quantiles.

To obtain a deeper understanding of causal shapes, we also performed piecewise MR using the LACE estimates to investigate whether any of the relationships had a threshold effect and to confirm the results of the non-linearity tests. Unlike fractional polynomial MR, this method does not smooth over the different quantiles. Instead, it fits a linear model in each quantile, with the slope representing the LACE. For each trait, we also conducted sex-stratified analysis. All analyses were performed in R versions 3.6.2 and 4.3.2 (https://www.R-project.org/).

### Sensitivity analyses

To address potential bias due to extreme values, varying incompleteness of phenotype data (e.g. LPA) and effects of factors such as menopause and waist–hip ratio, we performed several sensitivity analyses as follows: (1) using complete cases only; (2) excluding outliers of BMI, defined using Tukey’s lower and upper fences [29]; (3) including residuals from the first stage in the second stage (two-stage residual inclusion, 2SRI); (4) adjusting for lipid-lowering medication and waist–hip ratio; (5) excluding premenopausal or postmenopausal women, stratifying women by menopause status (self-reported or by age cut-point of 55 years), and stratifying both men and women by age; and (6) using a G-estimator method [30] to calculate causal estimates. All sensitivity analyses were also sex-stratified where applicable.

In 2SRI, the residuals are included as a control function to minimise bias of the standard 2SLS, especially when the effect measure is non-linear. The G-estimator gives a consistent estimate of the causal effect that varies the least. The causal effect estimates obtained using these methods should therefore not differ substantially from each other. We finally used two-sample MR to assess bidirectional causation.

## Results

Participants’ characteristics are shown in Table 1. The dataset included slightly more women (*n*=179,522, 53.8%) than men (*n*=154,060, 46.2%). On average, women had slightly lower BMI (27.0±5.1 kg/m^2^) compared with men (27.8±4.2 kg/m^2^). There was no difference in the mean age, men 57.1±8.1 years, women 56.7±7.9 years. Men had higher baseline mean BP (SBP=145±19.4 mmHg; DBP=86.6±11.0 mmHg) compared with women (SBP=138±21.2 mmHg; DBP=82.4±11.1 mmHg), and a higher prevalence of CMDs (e.g., 67.4% in men vs 32.6% in women for CAD). Differences in anthropometric measures and disease prevalence persisted across age groups (ESM Figs 2 and 3).

**Table 1:** Participant characteristics (*n*=333,582)

Characteristic	Men	Women
Proportion	46.2	53.8
Age (years)	57.1 (8.1)	56.7 (7.9)
BMI (kg/m^2^)	27.8 (4.2)	27.0 (5.1)
Townsend deprivation index	−1.59 (2.9)	−1.53 (3.0)
Smoking status		
Never	41.2	58.8
Previous	51.1	48.9
Current	53.7	46.3
Alcohol intake status		
Never	24.7	75.3
Previous	43.0	57.0
Current	46.8	53.2
Mortality	60.0	40.0
CMDs		
CAD	67.4	32.6
Type 2 diabetes	61.5	38.5
Stroke	61.3	38.7
CKD	55.2	44.8
Hypertension	53.7	46.3
Biomarkers		
SBP (mmHg)	145.0 (19.4)	138.0 (21.2)
DBP (mmHg)	86.6 (11.0)	82.4 (11.1)
Glucose (mmol/l)	5.2 (1.4)	5.1 (1.0)
HbA_1c_ (mmol/mol)	36.3 (7.3)	35.7 (5.7)
HbA_1c_ (%)	6.1 (1.9)	6.0 (1.7)
Cholesterol (mmol/l)	5.5 (1.1)	5.9 (1.1)
HDL-cholesterol (mmol/l)	1.3 (0.3)	1.6 (0.4)
LDL-cholesterol (mmol/l)	3.5 (0.9)	3.6 (0.9)
Triacylglycerols (mmol/l)	2.0 (1.2)	1.6 (0.9)
LPA (mmol/l)	43.4 (49.3)	44.6 (49.5)
Urea (mmol/l)	5.6 (1.4)	5.3 (1.3)

Continuous variables are presented as mean (SD) and categorical variables as percentages

In the 2SLS analyses, BMI was associated with type 2 diabetes (OR 3.10; 95% CI 2.73, 3.53; *p*=1.38×10^−67^), hypertension (OR 1.53; 95% CI 1.44, 1.62; *p*=8.92×10^−44^) and CAD (OR 1.20; 95% CI 1.08, 1.33; *p*=6.86×10^−4^), but not CKD (OR 1.08; 95% CI 0.67, 1.72; *p*=0.76) or stroke (OR=1.08; 95% CI 0.92, 1.28; *p*=0.34) (Table 2).

**Table 2:** Estimates of causal relationships between BMI and cardiometabolic outcomes using 2SLS MR in the UKB

	Combined		Men		Women	
Trait	OR/*β* (95% CI)	*p* value	OR/*β* (95% CI)	*p* value	OR/*β* (95% CI)	*p* value
CMDs						
CAD	1.20 (1.08, 1.33)	6.86 × 10^−4^	1.30 (1.15, 1.47)	2.55 × 10^−5^	0.97 (0.81, 1.18)	0.78
Type 2 diabetes	3.10 (2.73, 3.53)	1.38 × 10^−67^	2.85 (2.43, 3.33)	2.61 × 10^−38^	3.51 (2.84, 4.33)	2.99 × 10^−31^
Stroke	1.08 (0.92, 1.28)	0.34	1.14 (0.92, 1.40)	0.23	1.00 (0.77, 1.30)	0.98
CKD	1.08 (0.67, 1.72)	0.76	1.13 (0.62, 2.06)	0.69	0.99 (0.47, 2.06)	0.97
Hypertension	1.53 (1.44, 1.62)	8.92 × 10^−44^	1.50 (1.38, 1.63)	1.49 × 10^−22^	1.55 (1.42, 1.70)	9.28 × 10^−23^
Biomarkers						
DBP	0.15 (0.12, 0.19)	1.30 × 10^−18^	0.13 (0.09, 0.18)	4.91 × 10^−8^	0.17 (0.12, 0.22)	7.25 × 10^−12^
SBP	0.09 (0.06, 0.12)	2.31 × 10^−7^	0.10 (0.06, 0.15)	2.71 × 10^−5^	0.07 (0.03, 0.12)	2.37 × 10^−3^
Glucose	0.16 (0.13, 0.20)	4.90 × 10^−24^	0.18 (0.13, 0.23)	6.76 × 10^−12^	0.15 (0.10, 0.20)	7.77 × 10^−9^
HbA_1c_	0.22 (0.19, 0.26)	2.30 × 10^−34^	0.23 (0.18, 0.28)	3.85 × 10^−18^	0.22 (0.17, 0.27)	4.09 × 10^−18^
Cholesterol	−0.18 (−0.21, −0.14)	1.37 × 10^−24^	−0.23 (−0.28, −0.18)	3.96 × 10^−19^	−0.13 (−0.18, −0.08)	5.13 × 10^−8^
HDL-cholesterol	−0.26 (−0.30, −0.22)	4.36 × 10^−35^	−0.32 (−0.37, −0.26)	3.66 × 10^−28^	−0.25 (−0.31, −0.20)	5.69 × 10^−19^
LDL-cholesterol	−0.10 (−0.14, −0.07)	9.59 × 10^−10^	−0.17 (−0.21, −0.12)	4.79 × 10^−11^	−0.05 (−0.09, 0.00)	0.05
Triacylglycerols	0.13 (0.09, 0.16)	2.38 × 10^−13^	0.14 (0.09, 0.18)	3.76 × 10^−8^	0.12 (0.07, 0.17)	1.82 × 10^−6^
LPA	0.02 (−0.02, 0.05)	0.31	0.01 (−0.05, 0.05)	0.99	0.04 (−0.01, 0.09)	0.16
Urea	0.05 (0.01, 0.08)	0.01	0.02 (−0.03, 0.07)	0.37	0.06 (0.02, 0.11)	8.70 × 10^−3^

For disease-risk biomarkers (coefficients expressed in SD units), urea (*β*=0.05; 95% CI 0.01, 0.08; *p*=0.01) and LPA levels (*β*=0.02; 95% CI −0.02, 0.05, *p*=0.31) were not significantly associated with BMI, after correcting for multiple testing (*p*_Bonferroni_=0.001). A positive causal effect of BMI was observed for glucose (*β*=0.16; 95% CI 0.13, 0.20; *p*=4.90×10^−24^), HbA_1c_ (*β*=0.22; 95% CI 0.19, 0.26, *p*=2.30×10^−34^), and triacylglycerol levels (*β*=0.13; 95% CI 0.09, 0.16, *p*=2.38×10^−13^). BMI had an inverse causal relationship with total cholesterol (*β*=−0.18; 95% CI −0.21, −0.14, *p*=1.37×10^−24^), LDL-cholesterol (*β*=−0.10; 95% CI −0.14, −0.07, *p*=9.59×10^−10^) and HDL-cholesterol (*β*=−0.26; 95% CI −0.30, −0.22, *p*=4.36×10^−35^). The effect of BMI on DBP variation (*β*=0.15; 95% CI 0.12, 0.19, *p*=1.30×10^−18^) was almost twice the effect on SBP variation (*β*=0.09; 95% CI 0.06, 0.12, *p*=2.31×10^−7^) (Table 2).

### Sex-stratified analyses

As shown in Table 2, the causal effect of BMI on CAD in women was not statistically significant (OR=0.97; 95% CI 0.81, 1.18, *p*=0.78), but it was in men (OR=1.30; 95% CI 1.15, 1.47, *p*=2.55×10^−5^) (*p* value for sex difference=0.01; however, this was not significant after accounting for multiple testing, *p*<0.001, Table 3). No significant differences between sexes were observed for the causal effects of BMI on type 2 diabetes, stroke, hypertension or CKD.

**Table 3:** Cochran’s *Q* test of the difference between men and women for causal effects of BMI on cardiometabolic traits

Trait	Men vs all women	Men vs premenopausal women	Men vs postmenopausal women
CMDs			
CAD	0.011	0.115	0.006
Type 2 diabetes	0.121	0.325	0.541
Stroke	0.440	0.434	0.139
CKD	0.783	0.615	0.570
Hypertension	0.578	0.003	0.542
Biomarkers			
DBP	0.333	0.083	0.710
SBP	0.369	0.102	0.157
Glucose	0.355	0.729	0.098
HbA_1c_	0.893	0.286	0.481
Cholesterol	0.005	0.001	0.067
HDL-cholesterol	0.111	0.913	0.009
LDL-cholesterol	5.94 × 10^−4^	2.31 × 10^−4^	0.032
Triacylglycerols	0.600	0.136	0.077
LPA	0.328	0.897	0.201
Urea	0.239	0.268	0.467

Of the biomarkers, LDL-cholesterol was not significantly associated with BMI in women (*β*=−0.05; 95% CI −0.09, 0.00, *p*=0.05), but it was in men (*β*=−0.17; 95% CI −0.21, −0.12, *p*=4.79×10^−11^), and this sex difference was significant even after adjusting for multiple testing (*p*_Bonferroni_=0.001). The causal effect of BMI on total cholesterol in men (*β*=−0.23; 95% CI −0.28, −0.18, *p*=3.96×10^−19^), was almost double the effect seen in women (*β*=−0.13, 95% CI −0.18, −0.08, *p*=5.13×10^−8^), but the sex difference did not persist after correcting for multiple testing (*p*_Bonferroni_=0.001). In men, urea was not significantly associated with BMI (*β*=0.02; 95% CI −0.03, 0.07, *p*=0.37); however, a positive association was observed in women (*β*=0.06; 95% CI 0.02, 0.11, *p*=8.70×10^−3^), although this was not significant after Bonferroni correction. BMI was associated with DBP in both men and women, but was associated with SBP in men only (Tables 2 and 3).

### Effect of BMI on any cardiometabolic disease outcome

**Fig. 1:**
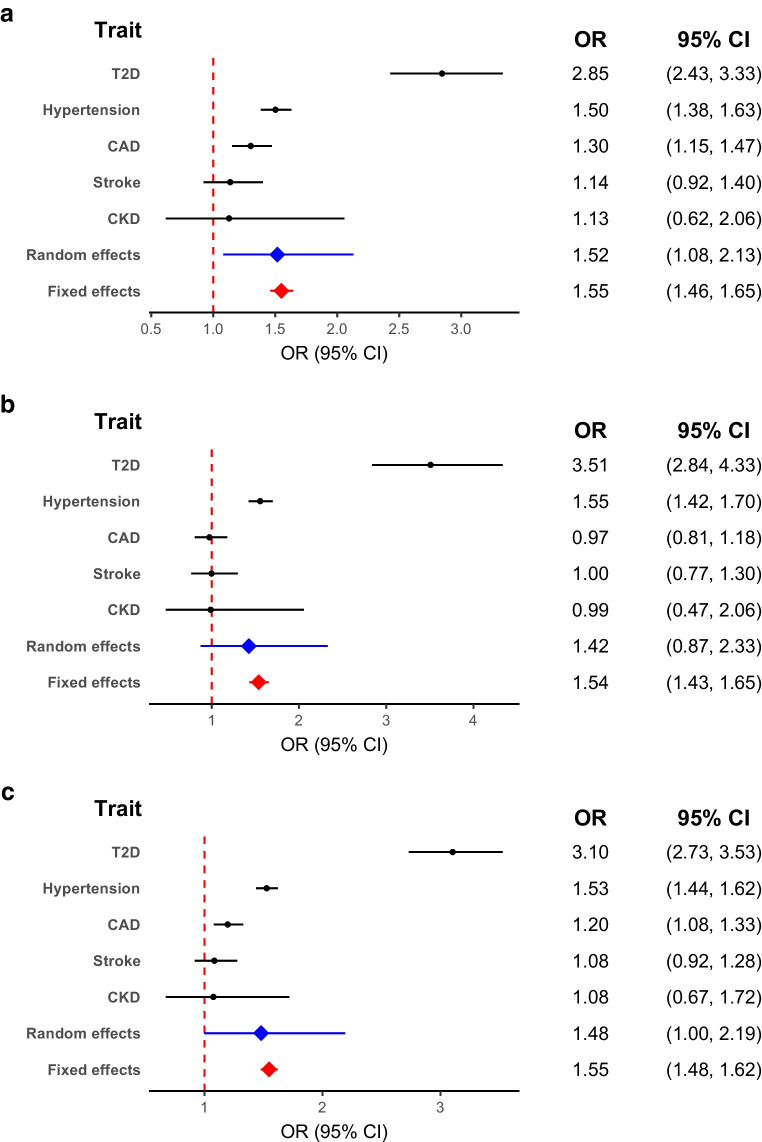
Forest plots of a summary meta-analysis combining the causal effect estimates of BMI on CMDs in (**a**) men, (**b**) women, and (**c**) all participants. The common outcome in both fixed and random effect lines represents any CMD. T2D, type 2 diabetes

In combined meta-analysis of the causal effect sizes of BMI on CMD, BMI was significantly associated with increased causal odds of any CMD (fixed effects OR 1.55; 95% CI 1.48, 1.62; *p*=8.23×10^−78^; random effects OR 1.48; 95% CI 1.00, 2.19; *p*=0.05). In men, BMI was also causally linked to any CMD using both fixed and random effects, but in women this association was only significant when considering fixed effects (Fig. 1).

### 2SLS sensitivity analyses

In the combined analyses, results did not differ across the three methods used to estimate causal effects (2SLS, 2SRI and G-estimator) for any trait except BP, HbA_1c_ and LPA levels, where use of the G-estimator gave larger effect sizes with wider 95% CIs (ESM Tables 3 and 4). Adjusting for lipid-lowering medication or waist–hip ratio did not materially change the results in either the main analysis or when excluding outliers for BMI (ESM Figs 4 and 5; ESM Tables 5 and 6).

A sex difference in effects of BMI on hypertension was observed when comparing men to premenopausal women, but this was not significant after accounting for multiple testing (*p*_Bonferroni_=0.001). Significant sex differences were observed for the relationship between BMI and LDL-cholesterol after multiple testing correction, but not when comparing men to postmenopausal women (ESM Table 7). In the age-stratified analyses (i.e., <55 years or 55 years and above), BMI was associated with CAD across all groups in men and in premenopausal (self-reported) women only. The causal effect of BMI was statistically significant in all groups for hypertension and type 2 diabetes, but not stroke or CKD (ESM Fig. 6 and ESM Table 8). Analyses performed to assess bidirectional causation did not yield results supporting such relationships. The association between SBP and BMI had a null effect size, while that between DBP and BMI suffered from horizontal pleiotropy (ESM Table 9).

### Shapes of causal relationships

**Fig. 2:**
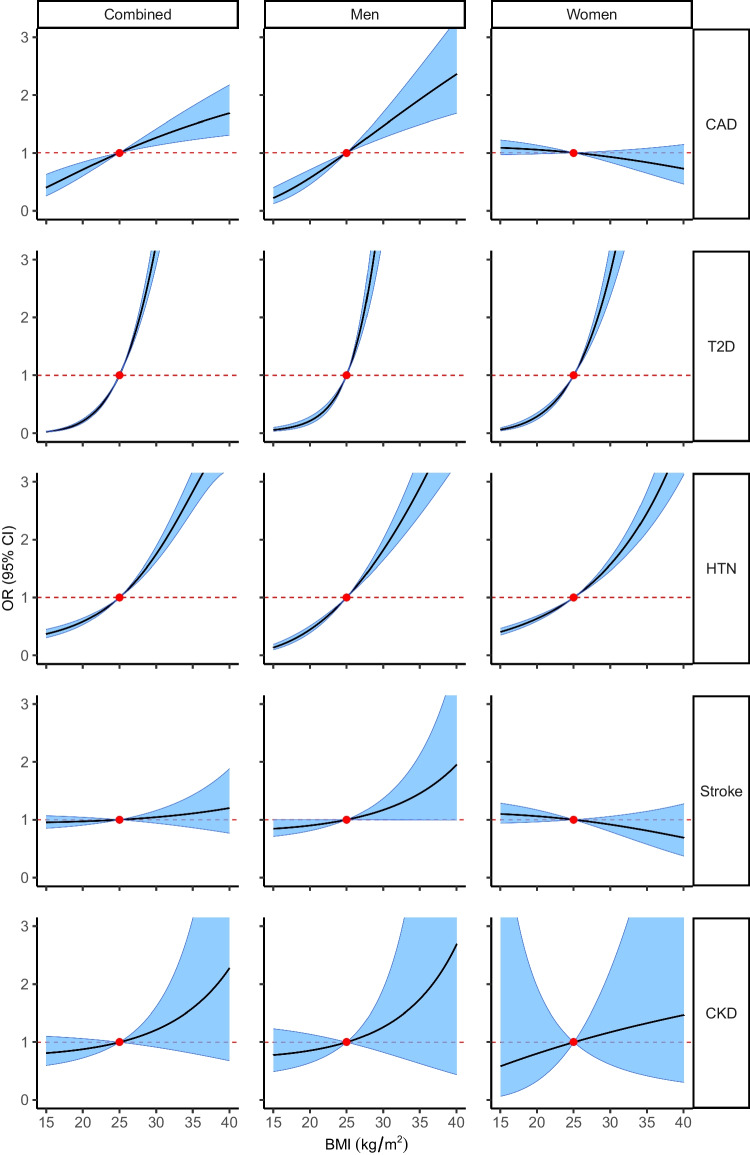
Plots showing the estimated shapes of the causal relationships between BMI and CMDs in combined and sex-specific analyses. Shape estimates are derived from the function of fractional polynomials based on the doubly ranked method that best fits the data. The solid black line represents the function curve, the blue band represents the 95% CI, the red dot represents the reference BMI of 25 kg/m^2^, and the dashed red line represents the null effect size. The plots have been cropped to depict estimated causal associations up to an OR of 3.0 for ease of comparison. HTN, hypertension; T2D, type 2 diabetes

From the non-linear MR fractional polynomials (FP), there was evidence to support a non-linear causal effect of BMI on type 2 diabetes. A quadratic model (*p*_Quadratic_=9.45x10^–5^) and a fractional polynomial model (*p*_FP_=1.80x10^–4^) were both a better fit than a linear model. In sex-specific analyses, we found support for a non-linear relationship between BMI and type 2 diabetes only in men (*p*_Quadratic_ and *p*_FP_ <0.001). Nor was there evidence to suggest that BMI had a non-linear causal association with CKD in sex-stratified analyses (see Fig. 2).

**Fig. 3:**
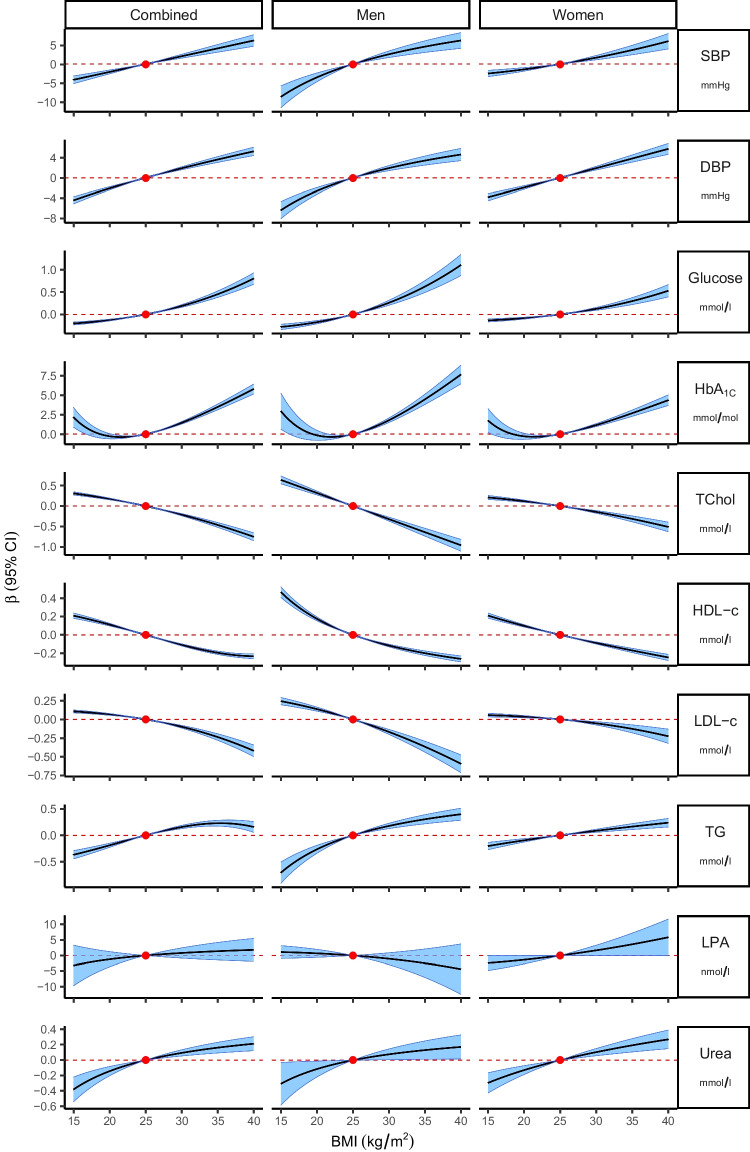
Plots showing the estimated shapes of the causal relationships between BMI and selected cardiometabolic biomarkers in combined and sex-specific analyses. Shape estimates are derived from the function of fractional polynomials based on the doubly ranked method that best fits the data. The solid black line represents the function curve, the blue band represents the 95% CI, the red dot represents the reference BMI of 25 kg/m^2^, and the red dashed line represents the null effect size. HDL-c, HDL-cholesterol; LDL-c, LDL-cholesterol; TChol, total cholesterol; TG, triacylglycerol

There was no statistically significant evidence to support a non-linear causal relationship between BMI and LPA, DBP, SBP and urea after correcting for multiple testing (*p*_Bonferroni_=0.001), but the results were significant for glycaemic and lipid biomarkers. When considering men and women separately, the data supported a non-linear causal association between BMI and HbA_1c_ in each group; and a non-linear causal association between BMI and HDL-cholesterol and triacylglycerols only in men (see Table 4 and Fig. 3). The piecewise MR results were consistent with these results; however, interpretation of the plots can be difficult, especially at the tails of the effect estimate distribution, where the linear segments are unrestricted and thus extrapolate to the most extreme values (ESM Fig. 7).

**Table 4:** Tests for shapes of causal relationships between BMI and cardiometabolic phenotypes derived from the residual and doubly ranked methods

Outcome	Subset	*p*_Q_		*p*_Quadratic_		*p*_FP_		*p*_FP degree_	
		Residual	Doubly ranked	Residual	Doubly ranked	Residual	Doubly ranked	Residual	Doubly ranked
CMDs									
CAD	Combined	0.36	0.13	0.02	6.07×10^–3^	0.10	0.05	0.18	0.09
	Women	0.00	0.13	0.28	0.04	0.43	0.29	0.44	0.11
	Men	0.44	0.59	0.08	0.14	0.10	0.11	0.83	0.92
T2D	Combined	0.60	0.06	4.25×10^–3^	9.45×10^–5^	0.01	1.80×10^–4^	0.28	0.45
	Women	0.02	0.75	0.08	0.22	0.17	0.20	0.10	0.93
	Men	0.73	8.80×10^–4^	0.02	5.47×10^–6^	0.05	5.53×10^–5^	0.21	0.03
HTN	Combined	0.73	2.17×10^–3^	0.83	0.06	1.00	0.15	0.15	0.03
	Women	0.03	0.14	0.08	0.82	0.08	1.00	0.20	0.27
	Men	0.21	0.41	0.10	0.02	0.23	0.02	0.27	0.78
Stroke	Combined	0.10	0.02	0.79	0.92	0.74	0.90	0.99	0.99
	Women	0.51	0.12	0.55	0.11	0.70	0.38	0.54	0.32
	Men	0.75	0.44	0.34	0.18	0.44	0.32	0.85	0.60
CKD	Combined	0.51	0.64	0.50	0.53	0.44	0.61	0.26	0.70
	Women	0.00	0.61	0.01	0.83	4.68×10^–3^	0.90	1.00	0.95
	Men	0.00	0.14	0.06	0.51	0.01	0.64	0.06	0.61
Stroke	Combined	0.10	0.02	0.79	0.92	0.74	0.90	0.99	0.99
	Women	0.51	0.12	0.55	0.11	0.70	0.38	0.54	0.32
	Men	0.75	0.44	0.34	0.18	0.44	0.32	0.85	0.60
Biomarkers									
DBP	Combined	0.24	0.17	3.33×10^–3^	0.21	9.12×10^–3^	0.30	0.38	0.32
	Women	0.40	0.13	0.31	0.99	0.46	1.00	0.34	0.46
	Men	0.24	0.70	1.12×10^–3^	0.11	1.36×10^–3^	0.11	0.61	0.98
SBP	Combined	0.48	0.16	0.04	0.93	0.04	1.00	0.97	0.60
	Women	0.22	0.41	0.56	0.66	0.60	0.82	0.90	0.38
	Men	0.48	0.64	0.02	0.22	9.36×10^–3^	0.21	0.96	0.99
Glucose	Combined	0.24	9.04×10^–4^	2.17×10^–5^	1.40×10^–5^	2.16×10^–4^	3.46×10^–5^	0.02	0.18
	Women	0.67	0.48	0.22	0.03	0.25	0.03	0.82	0.81
	Men	0.39	1.31×10^–5^	1.32×10^–3^	2.17×10^–4^	2.34×10^–3^	9.05×10^–4^	0.01	0.06
HBA_1c_	Combined	1.38×10^–4^	6.68×10^–10^	9.54×10^–10^	4.12×10^–12^	7.25×10^–8^	7.46×10^–11^	3.27×10^–3^	1.86×10^–3^
	Women	0.00	2.79×10^–3^	3.11×10^–4^	5.27×10^–5^	9.13×10^–4^	1.31×10^–4^	0.33	0.02
	Men	0.04	1.45×10^–6^	3.69×10^–7^	1.54×10^–7^	2.77×10^–5^	1.82×10^–6^	0.01	0.03
Total cholesterol	Combined	5.68×10^–5^	0.02	2.50×10^–10^	6.73×10^–3^	3.42×10^–8^	7.98×10^–3^	6.75×10^–3^	0.59
	Women	0.23	0.18	3.47×10^–5^	0.10	1.55×10^–3^	0.11	0.02	0.86
	Men	0.01	0.03	4.95×10^–5^	0.50	1.86×10^–4^	1.00	0.09	0.14
HDL-c	Combined	0.02	9.06×10^–5^	7.04×10^–8^	1.63×10^–6^	1.82×10^–6^	1.83×10^–5^	0.03	9.04×10^–3^
	Women	0.19	0.37	1.62×10^–4^	0.10	2.31×10^–3^	0.15	0.01	0.46
	Men	0.02	0.04	2.45×10^–5^	1.07×10^–4^	3.02×10^–5^	2.08×10^–4^	0.62	0.52
LDL-c	Combined	7.94×10^–4^	5.17×10^–6^	2.56×10^–13^	7.76×10^–6^	2.78×10^–5^	4.00×10^–5^	9.00×10^–9^	0.21
	Women	0.11	0.02	1.91×10^–6^	1.90×10^–3^	9.09×10^–3^	0.01	2.64×10^–4^	0.07
	Men	0.09	0.05	4.41×10^–6^	0.05	2.85×10^–3^	0.07	4.52×10^–3^	0.29
TG	Combined	2.61×10^–5^	1.04×10^–5^	2.36×10^–9^	8.82×10^–7^	4.07×10^–5^	1.44×10^–4^	8.76×10^–8^	2.54×10^–4^
	Women	0.34	0.48	2.36×10^–3^	0.34	0.05	0.48	1.24×10^–3^	0.45
	Men	0.12	3.02×10^–3^	3.34×10^–7^	6.37×10^–5^	1.16×10^–3^	8.84×10^–4^	8.05×10^–4^	0.08
LPA	Combined	0.74	0.57	0.07	0.50	0.47	0.59	0.97	0.90
	Women	0.74	0.68	0.51	0.65	0.60	0.68	0.86	0.84
	Men	0.75	0.16	0.07	0.06	0.55	0.40	0.16	0.24
Urea	Combined	0.48	0.30	3.33×10^–3^	0.06	2.28×10^–3^	0.05	0.50	0.63
	Women	0.96	0.03	0.43	0.47	0.23	0.45	0.75	0.96
	Men	0.13	0.34	1.43×10^–3^	0.10	0.04	0.19	0.05	0.30

*HDL-c* HDL-cholesterol, *HTN* hypertension, *LDL-c* LDL-cholesterol, *T2D* type 2 diabetes, *TG* triacylglycerol

## Discussion

In this study, we investigated the shapes of causal relationships between BMI, CMDs and biomarkers of disease risk. We further investigated sex differences within the causal framework, and estimated the causal effect of BMI on each CMD studied. The estimates from combined analyses showed that BMI is significantly associated with type 2 diabetes, CAD and hypertension, but not CKD or stroke; it is also associated with all assessed biomarkers except LPA and urea levels after controlling for multiple testing. In men, BMI associations mirrored those of the unstratified analyses, but BMI was not causally associated with CAD, LDL-cholesterol or SBP in women. BMI was causally associated with increased odds of any CMD in both sex-combined and sex-stratified analyses, when assuming fixed effects. When assuming random effects, the association in women was no longer significant. Sex differences persisted for causal effects of BMI on LDL-cholesterol only (with threefold attenuation of effect towards the null in women) after correcting for multiple testing. In investigations of non-linearity, after triangulation, the data support non-linear causal relationships between BMI and blood glucose levels, HbA_1c_ and all tested lipid fractions. In sex-stratified analyses, triangulated evidence supported a non-linear association between BMI and type 2 diabetes,  glucose, HDL-cholesterol and triacylglycerols only in men.

Causal associations between excess adiposity and cardiometabolic health have been reported previously, with results that are largely consistent with ours [13, 14, 31]. In our analysis, BMI was inversely associated with all cholesterol types and directly associated with triacylglycerol levels. This may reflect dyslipidaemic obesity, characterised by high levels of triacylglycerols and NEFAs, decreased HDL-cholesterol with HDL dysfunction (a shift towards proinflammation and altered reverse cholesterol transport), and normal or slightly increased LDL-cholesterol, attributed to altered metabolism favouring hypertriglyceridaemia [32]. One study assessed sex differences for causal effects of BMI in leading causes of death including cardiometabolic diseases such as type 2 diabetes, CAD and stroke [15]. In that study, BMI was causally related to the three diseases in men and women; the relationship with type 2 diabetes, but not CAD or stroke, varied by sex. In our study, BMI was not associated with stroke, and sex differences in type 2 diabetes were not replicated; the inconsistent findings between these studies may reflect our decision not to use sex-specific SNP effect estimates.

We found that BMI was associated with CAD in men but not all women. While some reasons for such findings may include weak instruments or violations of MR assumptions (conditional restriction), the ‘weak instrument test’ was not suggestive of weak instruments in our case (statistic=3015.08, *p*<2×10^−16^), and the Durbin–Wu–Hausman test supported the instrumental variable analysis as more consistent (*p*=1.04×10^−6^) than the ordinary least-squares regression. It is possible that a BMI PRS that is weighted using effect sizes from combined GWASs may not fully capture general adiposity in women, or that general adiposity itself is a poor predictor of CAD in all women. However, when women were grouped by menopause status, BMI was found to be significantly associated with CAD in premenopausal women only. This possibly points to detrimental effects of excess adiposity, which nullify the ‘protective effects’ of sex hormones [33]. In this cohort, premenopausal women with obesity had more than twice the prevalence of CAD compared with their non-obese counterparts (ESM Table 10). The null association observed in postmenopausal women may reflect dampened effects of general adiposity (or the presence of other competing/stronger risk factors) on CAD risk in this group. In two prospective studies of postmenopausal women, central or truncal obesity was associated with CAD/cardiovascular disease risk, but not general adiposity [34, 35]. We did not investigate different adiposity phenotypes, and the relationship between such phenotypes and CAD warrants further investigation within a causal framework.

Sexual dimorphism in lipid metabolism and the pathophysiology of CMDs is well-established [36–38]. For example, obesity tends to peak about 10 years earlier in men (50–54 years) compared with women (60–64 years). Even at the same BMI and age or fitness levels, men have a worse cardiometabolic health profile despite women having higher fat mass and lower skeletal muscle mass [9, 36, 38, 39]. Women tend to store excess lipids in subcutaneous adipose tissue (which is considered to be protective against CMDs), especially in the gluteal–femoral region, while in men excess fat is more centrally distributed in the visceral adipose tissue (which increases risk of CMDs). These differences are diminished when perturbations in oestrogen levels occur, as in the menopause (low levels) or when taking oral contraceptives (supraphysiological levels) [38, 40].

While the observed sex differences do not stand after correcting for multiple testing, except for LDL-cholesterol, they are worth considering given the documented role of sexual dimorphism in energy homeostasis and cardiometabolic health. Further, despite mixed results from studies, sexual dimorphism may have implications for weight loss interventions in men and women with different levels of metabolic health [41–45]. Complications during pregnancy, such as gestational diabetes and pre-eclampsia, confer additional risk for CMDs in women. Furthermore, from our results, excess adiposity appears to be detrimental to women both pre- and post menopause, while men have a higher burden of CMD at an earlier age compared with women of similar BMI. Such differences may have clinical implications. For men, screening for CMD at an earlier age and at a lower BMI threshold could identify people predisposed to CMD earlier, who would benefit from timely interventions. In women, targeted screening for CMD should take into consideration obesity in premenopausal women.

Non-linear MR has been previously used to assess causal relationships (e.g., the effect of alcohol on cardiovascular disease [46] or BMI on socioeconomic status [47]), but there is a dearth of literature on the nature of the causal effects of BMI on cardiometabolic health between the sexes. In one study focused on CKD, BMI was found to be causally associated with CKD using summary data MR, with evidence of non-linearity in the UK Biobank using the residual method [48]. We found no such association in our analyses using the doubly ranked method. This may be partly explained by our selection of CKD cases, which was more detailed than the previous study (ESM Table 1). Determining the shapes of causal associations may help estimate the relative benefits of interventions at different levels of exposure. For instance, lowering BMI from 40 to 25 kg/m^2^ would result in an approximately twofold decrease in the causal risk of type 2 diabetes (Fig. 2). Use of causal estimates could therefore provide a powerful tool for public health decision-making.

Causal inference studies using MR attempt to give an unbiased estimate of a causal effect of a given exposure on an outcome of interest, provided that the assumptions of MR are not violated, and the instruments explain sufficient variance in the exposures and/or outcomes of interest. To mitigate potential bias, we specifically used SNPs generated from GWASs that did not overlap with the UK Biobank, as the latter dataset was used in our primary analyses. We also performed sensitivity analyses to assess whether the results would change, and chose SNPs unrelated to each specific outcome to mitigate chances of horizontal pleiotropy. Although other problems of MR, such as canalisation, cannot be formally assessed, we believe that the estimates provided in this study offer a glimpse into the differences in causal effects of BMI on CMD between men and women, supporting further investigation.

### Strengths

In this study, we used MR, which offers a powerful alternative to assess causal relationships between exposures and outcomes of interest [49]. Conventional MR methods assume a linear relationship to estimate the population-averaged causal effect; however, we tested those linear assumptions to offer better insights for formulating public health policies and interventions [50]. Further, we used both residual and doubly-ranked non-linear MR and piece-wise linear MR to triangulate the evidence. We also had the advantage of a large sample size from the UK Biobank.

### Weaknesses

We used BMI as our sole measure of adiposity. BMI does not account for differential adiposity, nor is it a reliable measure of relative adiposity across different populations or ethnicities, making it hard to generalise the findings. However, BMI has been shown to be a reliable population-level measure for assessing general adiposity. MR faces challenges of horizontal pleiotropy and canalisation. While there is no formal method to test the latter, we selected BMI SNPs that were not associated with each respective outcome assessed, hence reducing the chances of horizontal pleiotropy. We also could not rule out methodological limitations, in that there may be other shapes, unavailable to us, that better fit these data. The field of MR is evolving quickly, and it may be that analyses need to be updated if and when there are fundamental changes in state-of-the-art in MR methods.

### Conclusion

In this analysis, BMI was found to be causally associated with increased risk of type 2 diabetes, CAD and hypertension, but not stroke or CKD, and was also associated with variation in disease-risk biomarkers, except LPA and urea. Further, BMI was causally associated with any CMD when considering fixed effects, in combined and sex-stratified analyses. We found evidence in support of a non-linear causal relationship between BMI and glycaemic and lipid biomarkers, except LPA. The adverse consequences of BMI on CAD risk are similar in men and premenopausal women. However, although BMI continues to confer increased CAD risk in men, it seems to be no longer a strong risk factor in postmenopausal women. These results further our understanding of the complex nature of the causal relationships between BMI and CMD. It also highlights the role of sex in CAD and lipid and glucose homeostasis in the context of causal risk conferred by excess adiposity, and underscores the need for consideration of sex in the management of excess adiposity. Finally, reducing excess adiposity remains highly beneficial in improving energy and lipid metabolism, as well as reducing the risk of CMD.

## Supplementary Information

ESM (PDF 846 kb)

## Data Availability

Individual-level data used in this study is available upon application to the UK Biobank. GWAS data used to compute polygenic risk scores are available at https://portals.broadinstitute.org/collaboration/giant/index.php/GIANT_consortium_data_files#GWAS_Anthropometric_2014_Height_Summary_Statistics

## Abbreviations

2SLS: Two-stage least-squares
2SRI: Two-stage residual inclusion
CAD: Coronary artery disease
CKD: Chronic kidney disease
CMD: Cardiometabolic disease
DBP: Diastolic blood pressure
GWAS: Genome-wide association study
LACE: Local average causal effect
LPA: Lipoprotein A
MR: Mendelian randomisation
PRS: Polygenic risk score
SBP: Systolic blood pressure
