# Correction: Investigating the causal relationships between excess adiposity and cardiometabolic health in men and women

**DOI:** 10.1007/s00125-024-06168-7

**Published:** 2024-07-01

**Authors:** Pascal M. Mutie, Hugo Pomares-Millan, Naeimeh Atabaki-Pasdar, Daniel Coral, Hugo Fitipaldi, Neli Tsereteli, Juan Fernandez Tajes, Paul W. Franks, Giuseppe N. Giordano

**Affiliations:** 1 Genetic and Molecular Epidemiology Unit, Lund University Diabetes Centre, Department of Clinical Sciences, Clinical Research Centre, Lund University, Lund, Sweden; 2 Harvard T.H. Chan School of Public Health, Boston, MA USA

**Correction: Diabetologia (2022) 66:321-335**

10.1007/s00125-022-05811-5

The original non-linear Mendelian randomisation approach used in this analysis, known as the ‘residual’ method [1], relies on the strong parametric assumptions of linearity and homogeneity between the genetic instrument and the exposure across strata of the exposure [2]. There have been reports that these assumptions were frequently violated [2, 3]. In response, the authors of the method [2] introduced the ‘doubly-ranked’ approach, implemented in a new R package, ‘SUMnlmr’ (https://github.com/amymariemason/SUMnlmr). We used this new approach to re-analyse our data, finding no evidence of non-linear causal relationships between BMI and chronic kidney disease (CKD) in either sex. We did find evidence of non-linear causal relationships between BMI and type 2 diabetes, blood glucose levels, HbA_1c_ and all tested lipid fractions in unstratified analyses. In sex-specific analyses, we found significant non-linear associations between BMI and HbA_1c_ in both sexes, and with type 2 diabetes, glucose, HDL-cholesterol and triacylglycerols only in men. The original article has been corrected to reflect the revised methodology; new data are shown in Figs 2 and 3, Table 4 and ESM Fig. 7.

In addition, the spelling of Hugo Pomares-Millan’s name has been corrected.
