# Supplementary material for: Investigating the causal relationships between excess adiposity and cardiometabolic health in men and women
Source: Diabetologia. 2022 Oct 12;66(2):321–35. doi: 10.1007/s00125-022-05811-5 (PMC9807546; doi:10.1007/s00125-022-05811-5)
Supplement: Supplementary file 1 — (PDF 846 kb) [file 125_2022_5811_MOESM1_ESM.pdf]

Electronic Supplementary Material (ESM)

Investigating the causal relationships between excess adiposity and cardiometabolic health in men and women

Pascal M. Mutie[1] (ORCID: 0000-0003-2626-1703), Hugo Pomares-Milan[1], Naeimeh Atabaki-Pasdar[1], Daniel Coral[1], Hugo Fitipaldi[1], Neli Tsereteli[1], Juan Fernandez Tajes[1], Paul W. Franks[1,2] (ORCID: 0000-0002-0520-7604), Giuseppe N. Giordano[1]

Table of Contents

ESM Tables *3*

ESM Table 1. Codes used for ascertaining disease outcomes in the UKB 3

ESM Table 2. UKB data field codes for biomarkers used 5

ESM Table 3. Sensitivity analyses of causal estimates of BMI on cardiometabolic outcomes in the UKB, combined analysis. 5

ESM Table 4. Sensitivity analyses of causal estimates of BMI on cardiometabolic outcomes in men and women in the UKB 6

ESM Table 5. Causal effect estimates of BMI adjusted for WHR on cardiometabolic outcomes 7

ESM Table 6. Causal effect estimates of BMI on cardiometabolic outcomes, adjusted for lipid-lowering medication 8

ESM Table 7. Estimates of causal effects of BMI on cardiometabolic outcomes in pre- and post-menopausal women in the UKB 9

ESM Table 8. Estimates of causal effect of BMI on main cardiometabolic diseases in different groups of men and women 10

ESM Table 9. Association between cardiometabolic traits and BMI (trait to BMI) 11

ESM Table 10. Prevalence of CAD among obese and non-obese premenopausal women 11

ESM Figures *12*

ESM Figure 1. 12

ESM Figure 2. 13

ESM Figure 3. 14

ESM Figure 4. 15

ESM Figure 5. 16

ESM Figure 6. 17

ESM Figure 7. 18

Abbreviations

2SLS	Two-stage least-squares
2SRI	Two-stage residual inclusion
CAD	Coronary artery disease
CKD	Chronic kidney disease
CMD	Cardiometabolic disease
DBP	Diastolic blood pressure
HTN	Hypertension
LPA	Lipoprotein A
MR	Mendelian randomisation
SBP	Systolic blood pressure

ESM Tables

ESM Table 1. Codes used for ascertaining disease outcomes in the UKB		
Type 2 diabetes	ICD10 codes	E110, E111, E112, E113, E114, E115, E116, E117, E118, E119, E136, E138, E139, E140, E141, E142, E143, E144, E145, E146, E147, E148, E149, E100, E101, E102, E103, E104, E105, E106, E107, E108, E109, E130, E131, E132, E133
	ICD9 codes	25000, 25010, 25020, 2503, 2504, 2505, 2506, 2507, 25090,25009,25019,25029,25099
	Self-reported codes	1220, 1223
	Medication codes (Insulin)	6153,6177
	Diabetes diagnosed by doctor (interview)	2443
	Age of diagnosis	2976 (excluded possible t1d by age <20yrs)
CAD	ICD10 codes	I200, I201, I208, I209, I210, I211, I212, I213, I214, I219, I220, I221, I228, I229, I231, I232, I233, I236, I238, I240, I241, I248, I249, I250, I251, I252, I253, I254, I255, I256, I258, I259
	ICD9 codes	4109,4119,4129,4140,4141,4141,4149
	Self-reported codes	1071
	OPCS4	K401, K402, K403, K404, K409, K411, K412, K413, K414, K424, K431, K433, K439, K441, K442, K449, K451, K452, K453, K454, K455, K456, K458, K459, K461, K462, K463, K464, K469, K472, K481, K482, K488, K491, K492, K493, K494, K498, K499, K501, K502, K503, K504, K508, K509, K751, K752, K753, K754, K758, K759
	OPCS4 self-reported	1070, 1095, 1523
	Vascular disease diagnosed by doctor	6150
Stroke	ICD10 codes	I600, I601, I602, I603, I604, I605, I606, I607, I608, I609, I610, I611, I612, I613, I614, I615, I616, I618, I619, I620, I621, I629, I630, I631, I632, I633, I634, I635, I636, I638, I639, I650, I651, I652, I653, I658, I659, I660, I661, I662, I663, I664, I668, I669, I64, I693, I694, H341, H342, H348, H349
	ICD9 codes	4309,4319,4340,4341,4349,4369
	Self-reported codes	1081, 1583,1491,1086
	OPCS4	A052, A053, A054, A103, L343, L353, L354
	OPCS4 self-reported	1468

	Vascular disease diagnosed by doctor	6150
Hypertension	ICD10 codes	I10, I110, I119, I120, I129, O10, O11, I130, I131, I132, I139, I150, I151, I152, I159
	ICD9 codes	4010,4011,4019,4020,4021,4029,4030,4031,4039,4040,4041,4049,4050,4051,4059
	Self-reported codes	1065,1072
	Vascular disease diagnosed by doctor	6150
	Medication (BP medication)	6153,6177
Kidney disease (CKD)	ICD10 codes	N180, N181, N182, N183, N184, N185, N188, N189, N19
	ICD9 codes	5859
	Self-reported codes	1193, 1192,1194
	OPCS4	M012, M013, M014, M015, M018, M019, M172, M174, L741, L742, L743, L744, L745, L746, L748, L749
	OPCS4 self-reported	1476, 1580,1581,1582,1195

ESM Table 2. UKB data field codes for biomarkers used	
Biomarker	UK biobank data field
Fasting glucose	30740
HbA1c	30750
Triglyceride levels	30870
Total cholesterol	30690
HDL	30760
LDL	30780
LPA	30790
Urea	30670
SBP	4080
DBP	4079

ESM Table 3. Sensitivity analyses of causal estimates of BMI on cardiometabolic outcomes in the UKB, combined analysis.

**Trait**	**2SLS-RI**		**G estimator**	
**CMDs**	**OR/β (95% CI)**	**P-value**	**OR/β (95% CI)**	**P-value**
CAD	1.19(1.07,1.32)	1.26x10^{-3}	1.19(1.07,1.32)	8.12 x10^{-4}
Type 2 diabetes	3.05(2.67,3.48)	8.77x10^{-62}	3.08(2.68,3.55)	2.68x10^{-55}
Stroke	1.08(0.92,1.27)	0.36	1.08(0.92,1.27)	0.34
CKD	1.07(0.67,1.71)	0.77	1.07(0.68,1.70)	0.76
Hypertension	1.54(1.45,1.64)	1.01x10^{-41}	1.55(1.45,1.64)	1.71x10^{-42}
Biomarkers				
DBP	0.16(0.12,0.19)	2.23x10^{-22}	1.60(0.82,2.39)	6.12x10^{-5}
SBP	0.09(0.06,0.12)	5.57x10^{-8}	1.63(0.29,2.97)	0.02
Glucose	0.16(0.13,0.20)	2.76x10^{-21}	0.18(0.12,0.25)	4.84x10^{-8}
HBA_{1c}	0.22(0.19,0.26)	7.27x10^{-39}	1.43(1.14,1.72)	2.88x10^{-22}
Cholesterol	-0.18(-0.21,-0.14)	9.59x10^{-25}	-0.21(-0.25,-0.16)	1.51x10^{-16}
HDL	-0.26(-0.3,-0.22)	2.91x10^{-42}	-0.10(-0.12,-0.08)	2.96x10^{-25}
LDL	-0.10(-0.14,-0.07)	1.15x10^{-9}	-0.09(-0.13,-0.06)	1.65x10^{-7}
Triacylglycerol	0.13(0.09,0.16)	1.09x10^{-14}	0.13(0.10,0.16)	8.70x10^{-17}
LPA	0.02(-0.02,0.05)	0.31	0.75(-0.99,2.50)	0.40
Urea	0.05(0.01,0.08)	0.01	0.07(0.01,0.12)	0.02

Effect sizes for CMDs are represented as OR. Effect sizes for biomarkers are represented as β SD units

ESM Table 4. Sensitivity analyses of causal estimates of BMI on cardiometabolic outcomes in men and women in the UKB								
	**2SLS-RI**				**G estimator**			
**Trait**	**Men**		**Women**		**Men**		**Women**	
**CMDs**	**OR/β (95% CI)**	***p* value**	**OR/β (95% CI)**	***p* value**	**OR/β (95% CI)**	***p* value**	**OR/β (95% CI)**	***p* value**
CAD	1.30(1.15,1.47)	3.42×10^{-5}	0.96(0.79,1.16)	0.65	1.30(1.15,1.47)	3.13×10^{-5}	0.98(0.82,1.16)	0.78
Type 2 diabetes	2.90(2.46,3.41)	7.13×10^{-37}	3.30(2.66,4.10)	4.59×10^{-27}	2.91(2.46,3.45)	4.85×10^{-35}	3.35(2.63,4.26)	7.34×10^{-23}
Stroke	1.14(0.92,1.40)	0.23	0.99(0.76,1.29)	0.94	1.14(0.92,1.40)	0.23	1.00(0.78,1.28)	0.98
CKD	1.13(0.62,2.05)	0.70	0.98(0.47,2.04)	0.96	1.13(0.62,2.05)	0.70	0.99(0.50,1.97)	0.97
Hypertension	1.53(1.40,1.67)	3.81×10^{-22}	1.56(1.42,1.71)	5.23×10^{-21}	1.53(1.41,1.67)	2.61×10^{-22}	1.56(1.42,1.71)	1.21×10^{-21}
Biomarkers								
DBP	0.14(0.09,0.18)	2.80×10^{-9}	0.17(0.13,0.21)	9.19×10^{-15}	1.63(0.52,2.75)	4.12×10^{-3}	1.52(0.42,2.61)	6.51×10^{-3}
SBP	0.11(0.06,0.15)	1.03×10^{-5}	0.07(0.03,0.12)	1.34×10^{-3}	2.27(0.38,4.16)	0.02	0.95(-0.94,2.84)	0.32
Glucose	0.18(0.13,0.23)	7.43×10^{-13}	0.15(0.1,0.19)	3.35×10^{-9}	0.25(0.16,0.35)	2.31×10^{-7}	0.12(0.03,0.21)	0.01
HBA_{1c}	0.23(0.18,0.27)	1.34×10^{-20}	0.22(0.17,0.27)	3.32×10^{-20}	1.62(1.18,2.05)	2.10×10^{-13}	1.26(0.87,1.65)	1.98×10^{-10}
Cholesterol	-0.23(-0.28,-0.18)	1.23×10^{-19}	-0.13(-0.18,-0.08)	5.30×10^{-8}	-0.23(-0.3,-0.17)	9.12×10^{-12}	-0.17(-0.24,-0.1)	9.30×10^{-7}
HDL	-0.32(-0.37,-0.27)	5.17×10^{-38}	-0.25(-0.3,-0.20)	7.22×10^{-24}	-0.1(-0.12,-0.07)	5.92×10^{-16}	-0.11(-0.14,-0.08)	7.30×10^{-13}
LDL[†]	-0.17(-0.21,-0.12)	4.59×10^{-11}	-0.05(-0.09,0.01)	0.05	-0.12(-0.17,-0.08)	4.10×10^{-7}	-0.06(-0.11,-0.01)	0.02
Triacylglycerol	0.14(0.09,0.18)	3.46×10^{-9}	0.11(0.07,0.16)	8.15×10^{-7}	0.17(0.12,0.21)	2.81×10^{-11}	0.09(0.06,0.13)	5.63×10^{-7}
LPA	0.01(-0.05,0.05)	0.99	0.04(-0.01,0.09)	0.16	-0.08(-2.56,2.4)	0.95	1.52(-0.93,3.97)	0.22
Urea	0.02(-0.03,0.07)	0.36	0.06(0.02,0.11)	9.10×10^{-3}	0.06(-0.02,0.14)	0.17	0.07(0.00,0.14)	0.06
Effect sizes for CMDs are represented as OR. Effect sizes for biomarkers are represented as β SD units								

ESM Table 5. Causal effect estimates of BMI adjusted for WHR on cardiometabolic outcomes						
**Trait**	**Combined**		**Men**		**Women**	
**CMDs**	**OR/β (95% CI)**	***p* value**	**OR/β (95% CI)**	***p* value**	**OR/β (95% CI)**	***p* value**
CAD	1.22(1.09, 1.37)	$7.53x10^{-4}$	1.36(1.18, 1.57)	$3.13 x10^{-5}$	0.97(0.79, 1.19)	0.78
Type 2 diabetes	3.56(3.07, 4.12)	$7.92 x10^{-65}$	3.4(2.81, 4.12)	$2.78 x10^{-36}$	3.87(3.07, 4.88)	$2.29 x10^{-30}$
Stroke	1.09(0.91, 1.31)	0.34	1.16(0.91, 1.48)	0.23	1(0.76, 1.33)	1.00
CKD	1.1(0.65, 1.85)	0.72	1.18(0.59, 2.37)	0.65	0.99(0.45, 2.16)	0.97
Hypertension	1.6(1.5, 1.71)	$3.76 x10^{-42}$	1.61(1.46, 1.77)	$1.35 x10^{-21}$	1.6(1.46, 1.76)	$4.53 x10^{-22}$
Biomarkers						
DBP	0.17(0.13, 0.21)	$1.93x10^{-19}$	0.16(0.1, 0.21)	$4.77 x10^{-8}$	0.18(0.13, 0.23)	$1.67 x10^{-12}$
SBP	0.1(0.06, 0.14)	$1.63 x10^{-7}$	0.12(0.06, 0.18)	$3.21 x10^{-5}$	0.08(0.03, 0.13)	$1.49 x10^{-3}$
Glucose	0.19(0.15, 0.22)	$3.86 x10^{-21}$	0.21(0.15, 0.27)	$3.43 x10^{-12}$	0.16(0.11, 0.21)	$1.54 x10^{-9}$
HBA_{1c}	0.25(0.21, 0.29)	$4.53 x10^{-34}$	0.26(0.2, 0.32)	$2.26 x10^{-17}$	0.24(0.18, 0.29)	$3.61 x10^{-18}$
Cholesterol	-0.2(-0.24, -0.16)	$6.39 x10^{-26}$	-0.27(-0.33, -0.21)	$5.52 x10^{-19}$	-0.14(-0.19, -0.09)	$2.33 x10^{-8}$
HDL	-0.29(-0.34, -0.24)	$2.34 x10^{-34}$	-0.37(-0.44, -0.3)	$4.06 x10^{-26}$	-0.28(-0.34, -0.22)	$2.70 x10^{-19}$
LDL	-0.12(-0.15, -0.08)	$2.88 x10^{-10}$	-0.19(-0.25, -0.13)	$6.63 x10^{-11}$	-0.05(-0.1, 0)	0.04
Triacylglycerol	0.14(0.1, 0.18)	$3.70 x10^{-14}$	0.16(0.1, 0.21)	$2.85 x10^{-8}$	0.13(0.08, 0.17)	$7.41 x10^{-7}$
LPA	0.02(-0.02, 0.06)	0.29	0(-0.06, 0.06)	0.99	0.04(-0.01, 0.09)	0.16
Urea	0.05(0.01, 0.09)	0.01	0.03(-0.03, 0.08)	0.36	0.07(0.02, 0.12)	$7.28 x10^{-3}$
Effect sizes for CMDs are represented as OR. Effect sizes for biomarkers are represented as β SD units						

ESM Table 6. Causal effect estimates of BMI on cardiometabolic outcomes, adjusted for lipid-lowering medication						
**Trait**	**Combined**		**Men**		**Women**	
CMDs	OR/β (95% CI)	***p*** value	OR/β (95% CI)	***p*** value	OR/β (95% CI)	***p*** value
CAD	1.2(1.08, 1.33)	$7.07x10^{-4}$	1.31(1.15, 1.48)	$2.81 x10^{-5}$	0.97(0.81, 1.17)	0.78
Type 2 diabetes	3.12(2.74, 3.55)	$4.68 x10^{-66}$	2.88(2.45, 3.39)	$2.50 x10^{-37}$	3.49(2.82, 4.32)	$1.20 x10^{-30}$
Stroke	1.08(0.92, 1.28)	0.34	1.14(0.92, 1.41)	0.23	1(0.77, 1.29)	0.98
CKD	1.08(0.67, 1.72)	0.76	1.13(0.62, 2.07)	0.69	0.99(0.48, 2.05)	0.97
Hypertension	1.53(1.44, 1.62)	$7.42 x10^{-43}$	1.51(1.39, 1.64)	$5.18 x10^{-22}$	1.55(1.42, 1.69)	$2.27 x10^{-22}$
Biomarkers						
Glucose	0.17(0.13, 0.2)	$2.33 x10^{-20}$	0.18(0.13, 0.23)	$5.00 x10^{-12}$	0.15(0.1, 0.2)	$5.47 x10^{-9}$
HBA_{1c}	0.22(0.19, 0.26)	$7.24 x10^{-35}$	0.23(0.18, 0.28)	$2.58 x10^{-18}$	0.22(0.17, 0.27)	$1.89 x10^{-18}$
HDL	-0.26(-0.3, -0.22)	$1.79x10^{-35}$	-0.32(-0.38, -0.27)	$3.16 x10^{-28}$	-0.25(-0.31, -0.2)	$2.56 x10^{-19}$
LDL	-0.11(-0.14, -0.07)	$6.91 x10^{-10}$	-0.17(-0.22, -0.12)	$3.93 x10^{-11}$	-0.05(-0.09, 0)	0.05
Triacylglycerol	0.13(0.09, 0.16)	$1.49 x10^{-13}$	0.14(0.09, 0.19)	$3.05 x10^{-8}$	0.12(0.07, 0.16)	$1.42 x10^{-6}$
LPA	0.02(-0.02, 0.05)	0.30	0(-0.05, 0.05)	1.00	0.04(-0.01, 0.09)	0.16
DBP	0.15(0.12, 0.19)	$4.95 x10^{-19}$	0.14(0.09, 0.18)	$3.93 x10^{-8}$	0.17(0.12, 0.22)	$4.23 x10^{-12}$
SBP	0.09(0.06, 0.12)	$1.79 x10^{-7}$	0.11(0.06, 0.15)	$2.37 x10^{-5}$	0.07(0.03, 0.12)	$2.11 x10^{-3}$
Urea	0.05(0.01, 0.08)	$9.54x10^{-3}$	0.02(-0.03, 0.07)	0.37	0.06(0.02, 0.11)	$7.88 x10^{-3}$
Cholesterol	-0.18(-0.21, -0.15)	$6.05 x10^{-25}$	-0.23(-0.29, -0.18)	$3.00 x10^{-19}$	-0.13(-0.18, -0.08)	$3.70 x10^{-8}$
Effect sizes for CMDs are represented as OR. Effect sizes for biomarkers are represented as β SD units						

ESM Table 7. Estimates of causal effects of BMI on cardiometabolic outcomes in pre- and post-menopausal women in the UKB				
**Trait**	Pre-menopause		Post-menopause	
**CMDs**	**OR/β (95% CI)**	***p* value**	**OR/β (95% CI)**	***p* value**
CAD	2.2(1.16, 4.18)	0.02	0.89(0.70, 1.14)	0.36
Type 2 diabetes	3.75(2.21, 6.36)	8.99 x10^{-7}	3.14(2.39, 4.13)	3.13x10^{-16}
Stroke	1.54(0.74, 3.2)	0.25	0.84(0.60, 1.18)	0.32
CKD	1.89(0.28, 12.87)	0.52	0.82(0.32, 2.09)	0.67
Hypertension	2.11(1.71, 2.59)	1.74 x10^{-12}	1.57(1.40, 1.76)	1.39 x10^{-14}
Biomarkers				
DBP	0.23(0.13, 0.33)	3.73 x10^{-6}	0.15(0.08, 0.22)	1.89 x10^{-5}
SBP	0.19(0.1, 0.29)	8.15 x10^{-5}	0.04(-0.02, 0.11)	0.20
Glucose	0.16(0.06, 0.26)	1.10 x10^{-3}	0.11(0.04, 0.18)	3.33 x10^{-3}
HBA_{1c}	0.29(0.19, 0.39)	1.38x10^{-8}	0.19(0.12, 0.27)	1.21 x10^{-7}
Cholesterol	-0.06(-0.15, 0.03)	0.18	-0.15(-0.22, -0.08)	2.31 x10^{-5}
HDL	-0.32(-0.44, -0.21)	9.92 x10^{-9}	-0.19(-0.27, -0.11)	1.55 x10^{-6}
LDL	0.03(-0.06, 0.12)	0.52	-0.07(-0.14, 0)	0.04
Triacylglycerol	0.22(0.12, 0.31)	7.99 x10^{-6}	0.06(-0.01, 0.13)	0.08
LPA	0.01(-0.09, 0.11)	0.88	0.06(-0.02, 0.14)	0.12
Urea	0.08(-0.01, 0.18)	0.08	0.06(-0.02, 0.13)	0.13
Effect sizes for CMDs are represented as OR. Effect sizes for biomarkers are represented as β SD units				

ESM Table 8. Estimates of causal effect of BMI on main cardiometabolic diseases in different groups of men and women								
Group	CAD		T2D		Hypertension		Stroke	
	OR(95% CI)	***p*** value	OR(95% CI)	***p*** value	OR(95% CI)	***p*** value	OR(95% CI)	***p*** value
All men	1.30(1.15,1.47)	$2.56x10^{-5}$	2.85(2.43,3.33)	$2.61x10^{-38}$	1.5(1.38,1.63)	$1.49x10^{-22}$	1.14(0.92,1.4)	0.23
All women	0.97(0.81,1.18)	0.78	3.51(2.84,4.33)	$2.99x10^{-31}$	1.55(1.42,1.7)	$9.28x10^{-23}$	1(0.77,1.3)	0.98
Pre-M women	2.20(1.16,4.18)	$1.58x10^{-2}$	3.75(2.21,6.36)	$8.99x10^{-7}$	2.11(1.71,2.59)	$1.74x10^{-12}$	1.54(0.74,3.2)	0.25
Post-M women	0.89(0.7,1.14)	0.36	3.14(2.39,4.13)	$3.13x10^{-16}$	1.57(1.4,1.76)	$1.39x10^{-14}$	0.84(0.6,1.18)	0.32
Younger women (<55 years)	1.47(0.95,2.28)	$8.65x10^{-2}$	4.31(2.98,6.25)	$1.10x10^{-14}$	1.73(1.49,2.02)	$1.34x10^{-12}$	1.28(0.78,2.09)	0.34
Older women (=/>55 years)	0.9(0.72,1.12)	0.35	3.43(2.62,4.48)	$2.27x10^{-19}$	1.55(1.38,1.74)	$7.14x10^{-14}$	0.93(0.67,1.28)	0.65
Pre-M, premenopausal; Post-M, postmenopausal								

ESM Table 9. Association between cardiometabolic traits and BMI (trait to BMI)			
**Trait**	**method**	***β***	***p* value**
CMDs			
CAD	Inverse variance weighted	0.98	0.393
	Egger Intercept	1.01	0.349
Type 2 diabetes	Inverse variance weighted	0.95	0.059
	Egger Intercept	1.01	0.039
Stroke	Inverse variance weighted	0.93	0.081
	Egger Intercept	1.00	0.956
CKD	Inverse variance weighted	0.99	0.737
	Egger Intercept	1.02	0.276
Hypertension	NA*	1.00	
Biomarkers			
SBP	Inverse variance weighted	0.00	0.001
	Egger Intercept	0.00	0.285
DBP	Inverse variance weighted	-0.01	0.000
	Egger Intercept	0.00	0.033
Glucose	Inverse variance weighted	-0.11	0.219
	Egger Intercept	0.01	0.443
HBA_{1c}	Inverse variance weighted	-0.01	0.657
	Egger Intercept	0.00	0.050
HDL	Inverse variance weighted	-0.03	0.124
	Egger Intercept	0.00	0.002
Triacylglycerol	Inverse variance weighted	-0.02	0.462
	Egger Intercept	0.00	0.166
Urea	Inverse variance weighted	0.03	0.637
	Egger Intercept	0.01	0.196
Cholesterol*	Inverse variance weighted	-0.06	0.041
LPA	NA^{Δ}		

$^{\Delta}$No SNPs remained after QC and harmonization. *Below optimum number of instruments after QC and harmonization). CAD = coronary artery disease, CKD = chronic kidney disease, T2D = type 2 diabetes, HBA_{1c} = glycated haemoglobin, LPA = lipoprotein(a), DBP = diastolic blood pressure, SBP = systolic blood pressure.

ESM Table 10. Prevalence of CAD among obese and non-obese premenopausal women			
Obesity	CAD	N	Frequency
No	No	36682	99.12 %
No	Yes	327	0.88 %
Yes	No	8993	97.89 %
Yes	Yes	194	2.11 %

ESM Figures

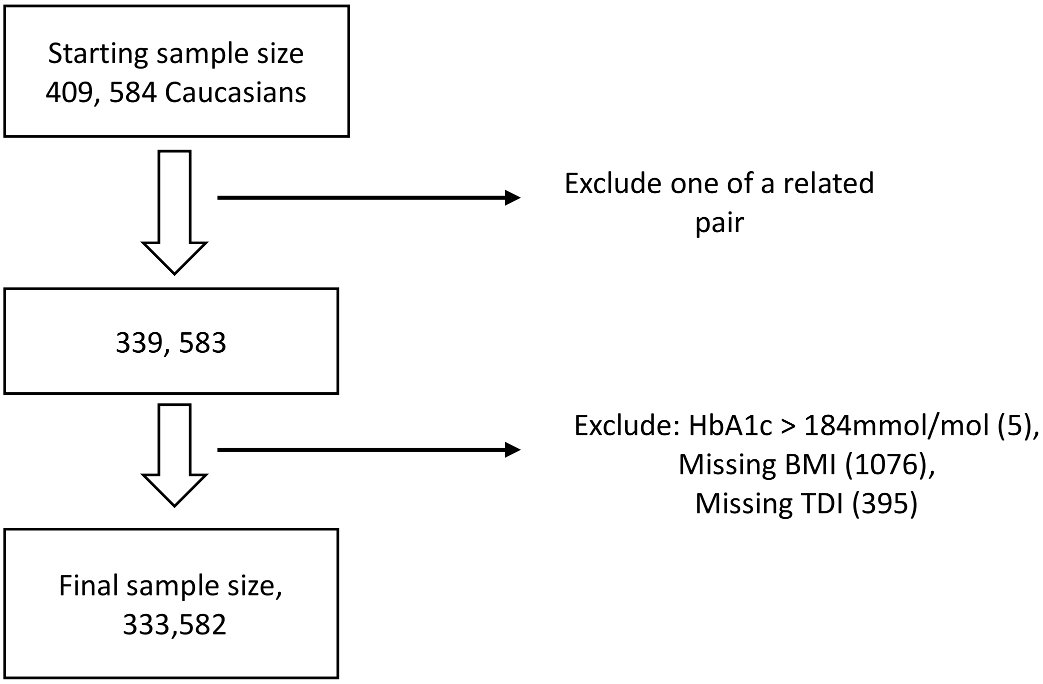

ESM Figure 1. Flow chart of final sample size ascertainment.

Prevalence of main cardiometabolic diseases in men and women.

In this cohort, men had a higher burden of disease compared to women and this burden persisted across all age groups after the youngest group where prevalence was the same except for hypertension. With increase in age, the prevalence of CAD and T2D increased, the highest prevalence being in men. Figure 2 below shows plots depicting prevalence for CAD, T2D, hypertension and stroke, in different age groups.

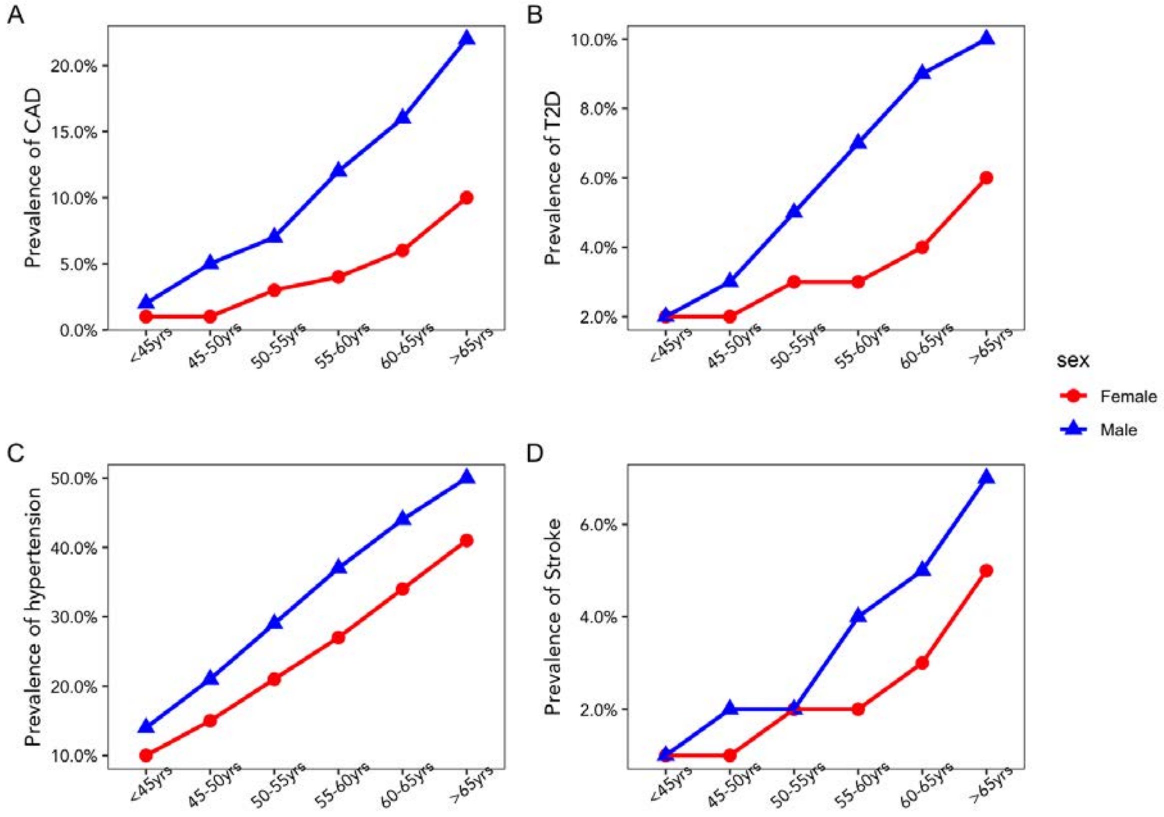

ESM Figure 2. Prevalence of main cardiometabolic diseases in men and women across different age groups in the UK Biobank.

BMI across age groups in men and women.
Men had a higher BMI than women across all age groups. The prevalence rose sharply up to ages 50-55 years, after which it plateaued in women and seemed to reduce slightly in men in the subsequent higher age groups. Figure 3.

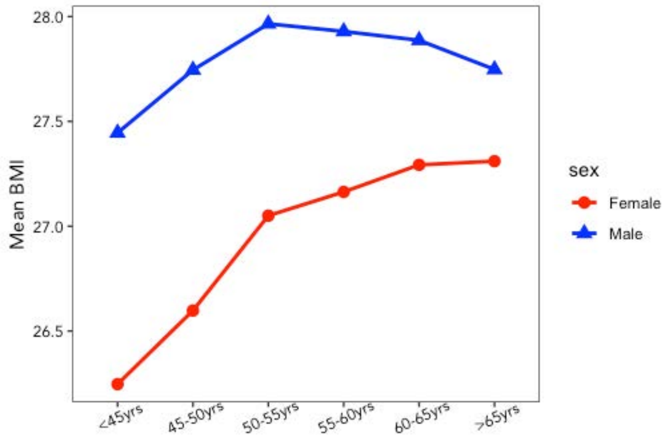

ESM Figure 3. Mean BMI (kg/m^2) in men and women across different age groups in the UK Biobank.

Sensitivity analysis: Adjusting for WHR, lipid-lowering medications and excluding outliers of BMI in each scenario.

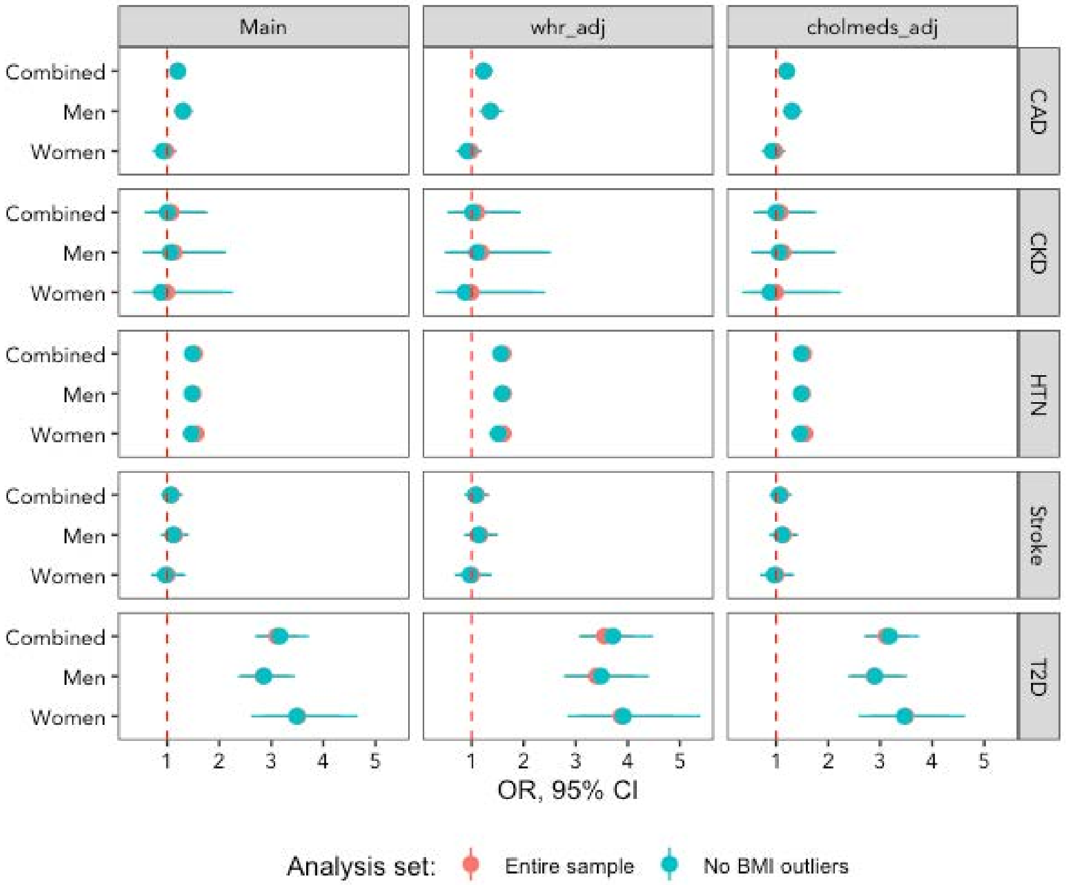

ESM Figure 4. Causal effect estimates of the effect of BMI on different cardiometabolic diseases, in men and women, comparing the main analysis with analyses accounting for waist-hip ratio (whr_adj) and lipid-lowering medication (cholmeds_adj) and also comparing the main dataset (red dots) and one excluding outliers of BMI (green dots) across all the adjustments. HTN = hypertension.

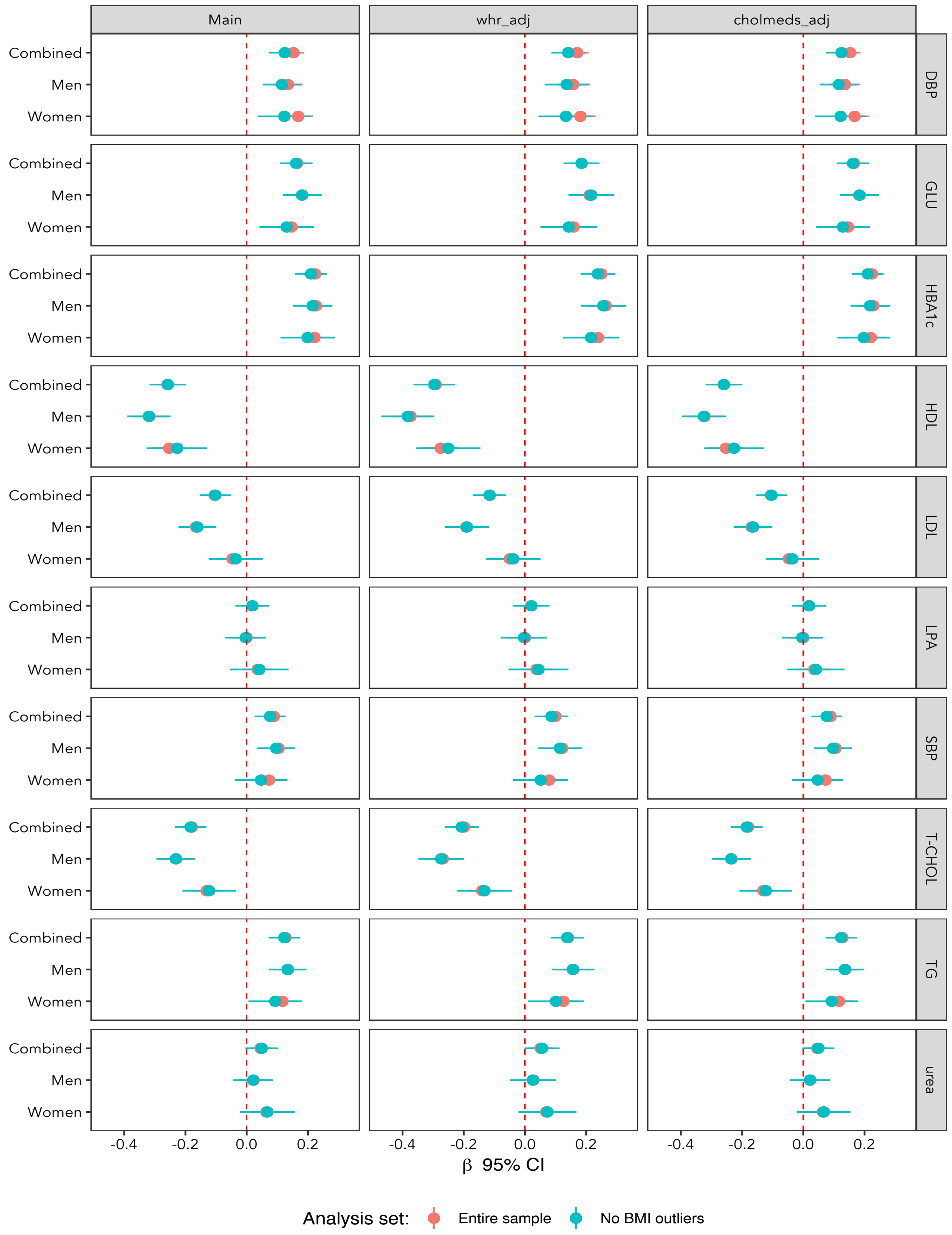

ESM Figure 5. Causal effect estimates of the effect of BMI on different cardiometabolic risk factors, in men and women, comparing the main analysis with analyses accounting for WHR (whr_adj) and lipid-lowering medication (cholmed_adj) and also comparing the main dataset (red dots) and one excluding outliers of BMI (green dots) across all the adjustments. T-CHOL = total cholesterol, GLU = glucose, TG = triacylglycerol, SBP = systolic blood pressure, DBP = diastolic blood pressure.

Effect of menopause and age on the causal association between BMI and cardiometabolic disease.

We performed analyses stratified by menopause status in women and by age in men and women and compared the results in the main disease outcomes. We excluded CKD because of the few case numbers which produced very wide confidence intervals and therefore unreliable results in these analyses.
BMI was associated with CAD in all age groups in men but in women the association was only observed in pre-menopausal women (self-reported). The association in younger women was positive but not significant. Association with stroke remained non-significant in all groups in men and women while associations with hypertension and T2D remained significant in all groups in men and women.

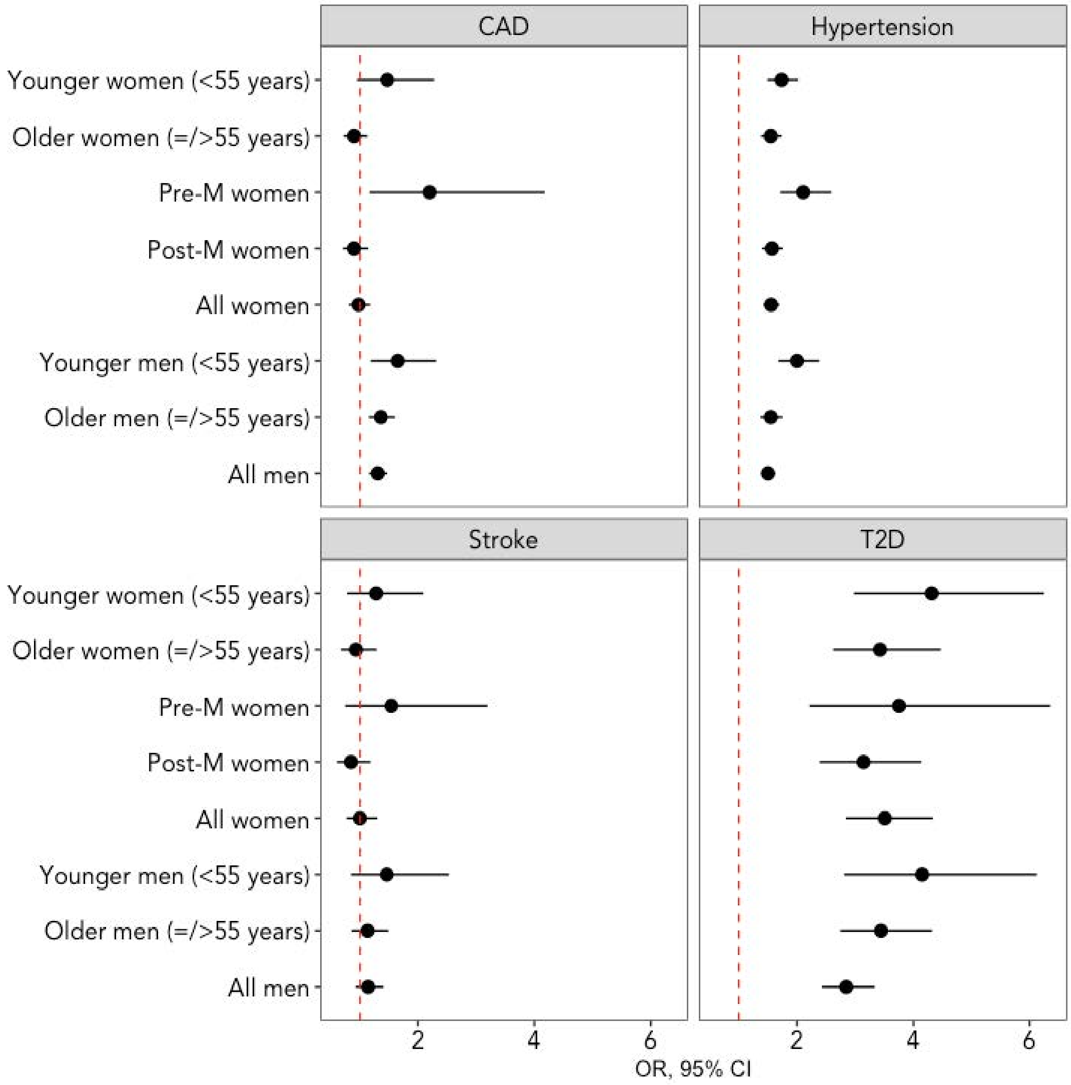

ESM Figure 6. Estimates of causal effect of BMI on main cardiometabolic diseases in different groups of men and women. Pre-M = premenopausal, Post-M = postmenopausal.

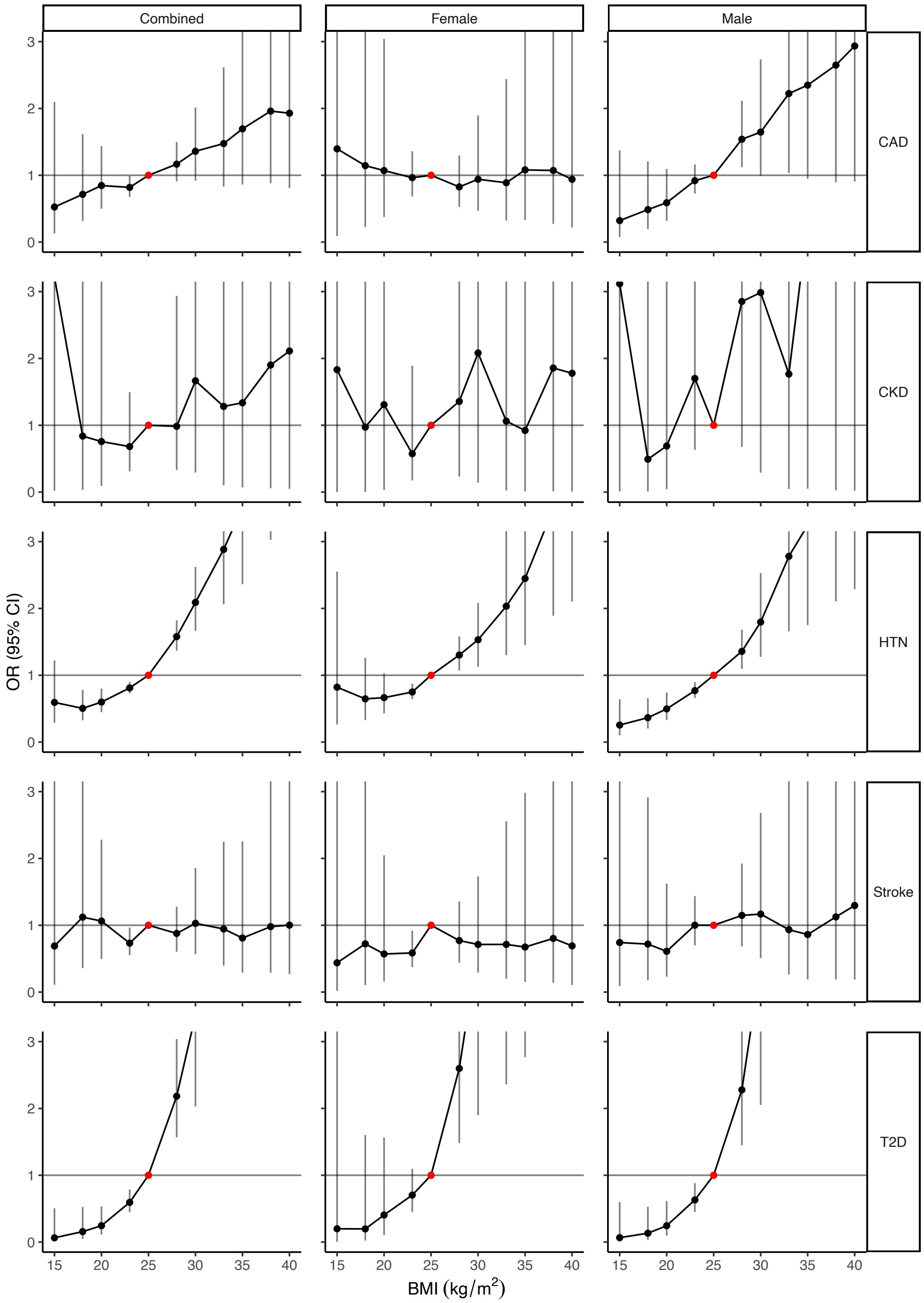

ESM Figure 7. Piecewise MR plots of estimated causal relationships between BMI and cardiometabolic diseases in combined and sex-specific analyses. The red dot represents reference BMI of 25kg/m^2 and the grey horizontal line represents the null effect size. Black dots represent quantile point estimates based on the quantile mean and the vertical grey lines represent the 95% CI. The X and Y axes have been truncated at 40 and OR of 3 respectively for ease of comparison.
